# Fenestrated carotid axis at the carotid bifurcation and long inferior petrosal sinus, novel findings

**DOI:** 10.1007/s00276-025-03730-9

**Published:** 2025-09-30

**Authors:** Mugurel Constantin Rusu, Viviana Dincă, Răzvan Costin Tudose

**Affiliations:** 1 Division of Anatomy, Faculty of Dentistry, “Carol Davila” University of Medicine and Pharmacy, 050474 Bucharest, Romania; 2 Research Department, “Dr. Carol Davila” Central Military Emergency Hospital, 010825 Bucharest, Romania

**Keywords:** Carotid artery, Dural venous sinus, Jugular vein, Parapharyngeal space, Anatomic variation

## Abstract

**Purpose:**

Fenestrations of the cervical carotid arteries are extraordinarily rare variants, and no previous evidence of the carotid bifurcation (CB) fenestration was reported. Also, the inferior petrosal sinuses (IPSs) with extracranial segments of different lengths are rare. Fenestrations of the IPSs were not reported previously. Such novel morphologies are reported here.

**Method:**

The anatomical variations described herein were detected during meticulous analysis of archived CT angiographic data obtained from a 45-year-old female subject.

**Results:**

On the left side, variants of the carotid system were found: an 8.42 mm long fenestration was observed on the CB, extending from the common to the internal carotid artery, with unequal limbs. The superior thyroid artery originated from the CB. Also, a 5.55 cm long extracranial segment of the IPS was detected along the internal jugular vein (IJV). They both appeared as a high, parapharyngeal duplication of the IJV. The extracranial IPS had a 9.01 mm long fenestration. The IJV was compressed between the C1 transverse process and the digastric muscle. A partially ossified 2.24 long left styloid process was observed.

**Conclusion:**

Fenestrated CB and, respectively, extracranial IPS, are novel risk morphologies for surgeons and interventionists that should be acknowledged and documented preoperatively.

**Supplementary Information:**

The online version contains supplementary material available at 10.1007/s00276-025-03730-9.

## Introduction

The common carotid artery (CCA) bifurcates (CB, carotid bifurcation) into the external (ECA) and internal (ICA) carotid arteries in the carotid triangle. The ICA ascends into the parapharyngeal space, enters the skull, and participates with branches at the circle of Willis to supply the brain. Carotid fenestration is extremely rare, with only a few case reports existing in the literature. However, although fenestrations of the ICA were reported previously, such anomalies were not found or reported in the CCA.

The inferior petrosal sinus (IPS) connects anteriorly to the cavernous sinus. Posteriorly, it exits through the antero-medial part of the jugular foramen to empty into the internal jugular vein (IJV) beneath that foramen. The IPS may continue adjacent to the IJV with an extracranial segment that may be regarded as an accessory IJV [1]. A long IPS empties into the IJV at a variable distance from the skull base [11]. Fenestrations of a long IPS were not reported previously.

## Material and method

A retrospective analysis was conducted on archived DICOM images from a 45-year-old female patient. The imaging study was performed as part of routine clinical care in a case where no pathological conditions were present that would alter the normal vascular architecture. This research adhered to the ethical standards outlined in the World Medical Association’s Declaration of Helsinki. Institutional approval was obtained from the responsible authorities (affiliation 2) under approval number 737/01 November 2024.

Imaging was acquired using a 32-slice CT scanner (Siemens Multislice Perspective Scanner, Forcheim, Germany) with 0.6 mm collimation and a 0.75 mm reconstruction slice thickness, with 50% overlap, to enable multiplanar maximum intensity projections. Image analysis was performed using Horos 3.3.6 software (Horos Project, Annapolis, MD, USA), as previously [5]. The anatomical findings were verified through both two-dimensional reconstructions and three-dimensional volume rendering techniques, with appropriate measurements obtained.

## Results

**Fig. 1:**
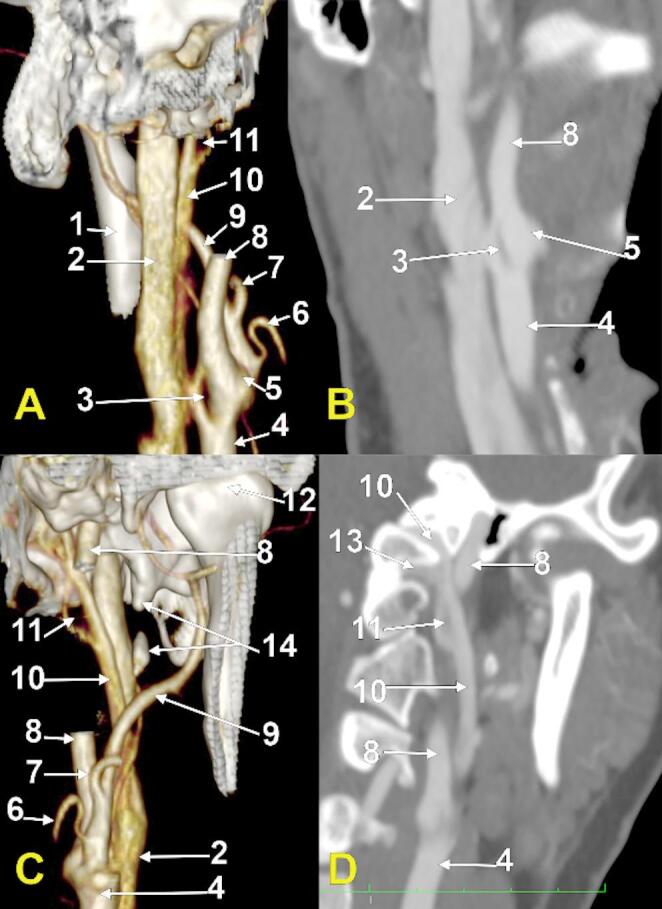
Fenestrated carotid axis at the carotid bifurcation (**A**, **B**). Fenestrated extracranial segment of a long inferior petrosal sinus (**C**, **D**). Left side. Three-dimensional renderings: A, posterior view; C, anterior view. Oblique coronal slices: B, posterior view; D, anterior view. (1) posterior margin of the mandibular ramus; (2) internal jugular vein; (3) fenestration of the carotid axis; (4) common carotid artery; (5) carotid bifurcation; (6) lingual artery; (7) facial artery; (8) internal carotid artery; (9) external carotid artery; (10) inferior petrosal sinus; 11. fenestration of the extracranial segment of the long inferior petrosal sinus; 12. head of the mandible; 13. anterior condylar vein; 14. partly ossified styloid process

Novel and rare morphological possibilities were found on the left side (Fig. 1). A 2.24 cm long left styloid process was found; it had a 4.17 mm unossified middle segment.

The left CB was at 0.35 cm postero-superior to the left hyoid tubercle. A longitudinal carotid fenestration of 8.42 mm/2.08 mm was found (Fig. 1A and B). It had a large antero-medial limb (4.91 mm) and a thin postero-lateral limb (2.16 mm). The postero-medial limb of the CCA’s fenestration was contacting and was partly hidden deep to the IJV. The upper 4.26 mm of that fenestration were on the ICA, while the lower 4.16 mm were on the CCA. At the limit between the CCA and the ICA, the superior thyroid artery originated from the CB (Online Resource 1).

The left IPS (Fig. 1C and D) left the posterior cranial fossa through the antero-medial part of the jugular foramen; the left IJV continued the sigmoid sinus in the postero-lateral part of the jugular foramen. A 5.55 cm long extracranial segment of the left IPS was found descending applied on the antero-medial side of the IJV. Immediately inferior to the jugular foramen, the anterior condylar vein joined the IPS. A fenestration of the IPS of 9.01/2.31 mm was found. Distally to that fenestration, the IPS was connected to the parapharyngeal plexus by a transverse vein passing between the ICA and ECA (Online Resource 1). Both the IPS and IJV were crossed laterally by the posterior belly of the digastric muscle; deep to this muscle coursed the occipital artery. The IJV was compressed between the transverse process of the atlas, medially, and the digastric muscle, laterally (Online Resource 1). Immediately distal to the fenestration of the IPS, the calibre of the extracranial segment of the IPS was 4.05 mm, and the IJV had a maximum diameter of 7.94 mm. The IPS and IJV appeared as a high duplication of the IJV in the parapharyngeal space.

## Discussion

Fenestrations of the ICA may occur in the cervical segment or its intracranial course [9, 10]. Fenestration, or duplication, of the cervical segment of the ICA is extremely rare [2, 3]. There is no clear embryologic mechanism that would explain the occurrence of a cervical ICA fenestration [2]. Gailloud et al. (2004) discussed that true ICA fenestrations are pseudo-fenestrations – arterial dissections with double lumen [2]. They argued that contour irregularities and size asymmetry of the limbs of the fenestration characterise such pseudo-fenestrations [2]. They also admitted that it is possible to hypothesise that a fenestration of the cervical ICA would itself be an anatomic variant predisposing to arterial dissection [2]. Killien et al. [4] reported a case of ICA duplication on its entire cervical portion. However, they stated that the CB was not involved. The precise division point is unclear and inconsistently illustrated [4]. Notably, their assessment relied on angiography, a technique that may overlook or misclassify arterial variations due to overlap and limited wall delineation. By definition, a duplication requires separate origins or a persistent bifurcation that does not reconstitute. In their case, the two lumina reunite before the petrous segment, which is diagnostic of a fenestration, not a true duplication [4].

We report here a carotid fenestration with asymmetric limbs located at the CB but lacking contour irregularities that would suggest an arterial dissection. Therefore, the CB fenestration may add to previously documented variations of it, such as the variable height and spin [5, 7]. The narrow limb of such a fenestration may predispose to thrombus formation and, therefore, may increase the risk of ischemic attacks in the cerebral territory of the ICA only, because the upper end of the fenestration limb in this case was inserted onto the ICA. As the narrow limb of the CB fenestration we report was inserted onto the CCA and ICA, it can be regarded as a variational bypass of the CB, which may be functionally useful. From a procedural standpoint, an unrecognized CB fenestration constitutes a risk morphology. In neck dissections, it increases haemorrhagic risk if mistaken for a single vessel. In carotid endarterectomy, it complicates clamp placement and arteriotomy planning, as adequate exposure of both limbs may be required. The narrow limb, in particular, could necessitate careful reconstruction. In carotid artery stenting, a fenestrated morphology could shorten landing zones, complicate stent apposition, and increase the risk of intimal injury or plaque displacement. During catheter-based diagnostics or interventions, inadvertent selection of the narrow limb may lead to endothelial trauma or distal embolization. Such risks increase if, as in this case, the superior thyroid artery, usually approached during thyroidectomies, originates from the CB and not from the ECA.

Endovascular access to the IPS has diagnostic and therapeutic utility for diverse conditions involving the cavernous sinus and sellar regions [8]. Therefore, the possibility of a long IPS should be checked before such interventions to avoid false paths or failures. Although scarce reports of long IPSs are available, the fenestration of its extracranial segment, reported here, is an absolutely novel morphology that interventionists should acknowledge. When the upper ends of a long IPS and IJV correlate with different skull base passages, the long IPS and IJV could be regarded as a high duplication of the IJV.

## Conclusions

This case demonstrates that rare fenestrations of the carotid bifurcation and IPS present significant clinical risks. The narrow limb of carotid fenestration may predispose to thrombus formation while simultaneously functioning as a natural bypass. Unrecognized fenestrations complicate surgical planning and increase haemorrhagic risk. A fenestrated extracranial IPS creates additional complexity for cavernous sinus interventions. These concurrent anatomical variants underscore the critical importance of comprehensive preoperative imaging to identify unexpected vascular duplications or fenestrations that alter procedural risk profiles. Surgeons and interventionalists must maintain awareness of these rare morphological possibilities to prevent procedural complications and ensure optimal patient outcomes.

## Supplementary Information

Below is the link to the electronic supplementary material.

Supplementary Material 1

## Data Availability

No datasets were generated or analysed during the current study.
