# Supplementary material for: Fenestrated carotid axis at the carotid bifurcation and long inferior petrosal sinus, novel findings
Source: Surg Radiol Anat. 2025 Sep 30;47(1):218. doi: 10.1007/s00276-025-03730-9 (PMC12484359; doi:10.1007/s00276-025-03730-9)
Supplement: Supplementary file 1 — Supplementary Material 1 [file 276_2025_3730_MOESM1_ESM.pdf]

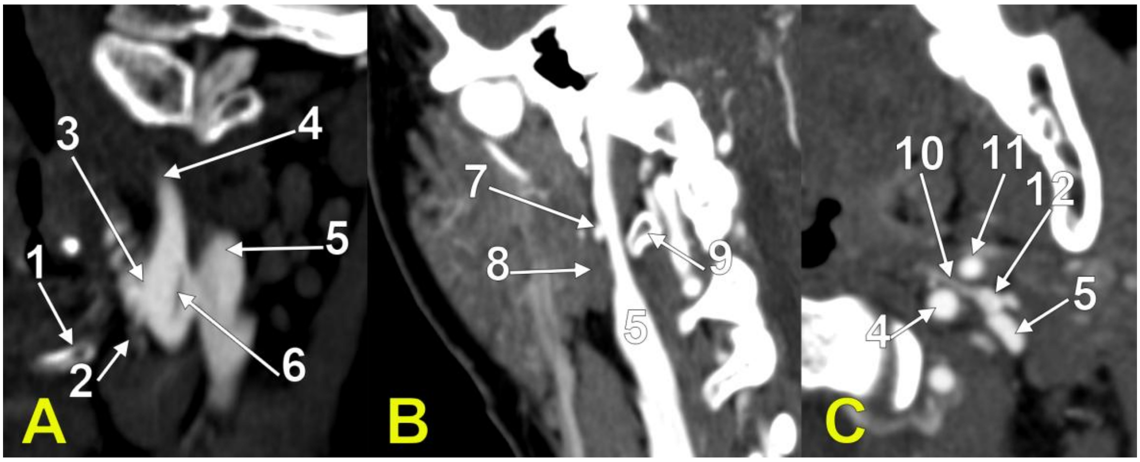

Details regarding the case. A. Oblique coronal slice through the left carotid bifurcation, anteriorly viewed. B. Oblique sagittal slice through the left internal jugular view, laterally viewed. C. Axial slice through the communicating vein (CV) between the inferior petrosal sinus and the parapharyngeal plexus. 1. greater hyoid horn; 2. superior thyroid artery; 3. carotid bifurcation; 4. internal carotid artery; 5. internal jugular vein; 6. carotid fenestration; 7. occipital artery; 8. digastric muscle; 9. transverse process of the atlas; 10. CV; 11. external carotid artery; 12. inferior petrosal sinus.
